# Adapting to Climate Change with *Opuntia*

**DOI:** 10.3390/plants12162907

**Published:** 2023-08-09

**Authors:** Ana O. S. Jorge, Anabela S. G. Costa, M. Beatriz P. P. Oliveira

**Affiliations:** LAQV@REQUIMTE, Department of Chemical Sciences, Faculdade de Farmácia, Universidade do Porto, R. Jorge Viterbo Ferreira 228, 4050-313 Porto, Portugal; up201706679@edu.ff.up.pt (A.O.S.J.); acosta@ff.up.pt (A.S.G.C.)

**Keywords:** prickly-pear, desertification, *Opuntia*, *Opuntia ficus-indica*, drought, arid soil, fodder

## Abstract

Adapting our food production chain and increasing the flora and fauna’s livelihood in climate change-affected areas using *Opuntia* is not only theoretical but already exists in practice in many places. This cactus grows in unsuitable soil for most species as it is adapted to arid and semi-arid soils and hot weather. In these regions, *Opuntia* protects from erosion and contributes to soil health. The usage of this plant as fodder is also discussed, with immense potential in substituting a part of livestock’s diet and even increasing the quality of the animal’s by-products and decreasing water consumption. This would result in a feed that is low-cost and has a lower environmental impact. It is to be noted that *Opuntia* has a high potential as an invasive species, with caution always being recommended when dealing with this specie. The high content of specific compounds, such as proline, indicaxanthin, and betanin, found in *Opuntia ficus-indica*, influence the plant’s adaptation to unfavourable conditions. This collective evidence depicts *Opuntia* as a crop that can battle climate change and ensure food security.

## 1. Introduction

*Opuntia ficus-indica* (L.) mill (prickly-pear cacti) is a fruit-bearing cactus native to Mexico, currently spread on all continents due to its outstanding resistance and propagation. The recent crisis in water availability further increased this plant’s territory, as it flourishes in semi-arid and arid regions. Due to its habitat preference and low water need, this plant is a suitable alternative to improve commercial income in these areas.

The *Opuntia* genus thrives in high temperatures, elevated levels of CO_2_ and low rainfall, all while having high biomass productivity and extensive root system growth [1]. The ideal temperature for *Opuntia* root growth is reported to be between 27 °C and 30 °C, while low temperatures (−16 °C) can damage the plant [2]. Temperatures of 2–6 °C are well tolerated by the plant, making it suitable to inhabit colder regions such as southern Europe [3]. In these regions, the most limiting factor to *Opuntia* growth is the winter’s low temperatures. These are reported by Snyman et al. [4] to have a negative impact on the plant’s moisture stress and phonological stage, although not causing death.

Regarding the plant’s usage for human consumption, all the plant parts are edible, including fruits, cladodes (*Opuntia* flattened stems), and flowers, which are used in infusions. This ensures low-waste crop cultivation, with all parts of the plant having potential. The cladodes have also been shown to be a good source of food for cattle [5]. *Opuntia* is highly palatable for cattle and is reported to improve the feed intake and productivity of sheep and dairy cows [6,7]. Its high content of digestible fibre and moisture is a significant source of nutrients for animals [1].

The consumption of this plant has been associated with several beneficial properties. This is due to its high content of digestible carbohydrates and antioxidant dietary fibre [8]; the capacity of some of its components to behave as radical scavengers [9]; its high content of minerals, antioxidants, and vitamin C [10]; and its seed oil being particularly rich in PUFAs which bring benefits to conditions such as coronary heart disease and rheumatoid arthritis [11], as well as having a high concentration in linoleic acid [12]. An example of a direct action of the consumption of the prickly pear fruit is the protective ability against ethanol-induced stomach ulcers [13]. Extracts nowadays have been proven effective in lowering cholesterol [14] and prevent type-II diabetes [15], and aqueous extracts are reported to inhibit in vitro growth of cancer cells [16]. Traditionally it has been used to treat various ailments. In Mexico, ancient tribes used *Opuntia* to cure superficial wounds and to enhance the immune system [17]. Traditional Chinese medicine suggests the usage of the cactus to treat inflation, relieve pain, and act as an antidote to snake venom [15]. In Sicily, prickly-pear flower extracts are known to have diuretic solid effects, and the cladodes are used as medicine for inflammation, edemas, and arthrosis [18].

Fresh consumption is not the only use of this cactus. The fruit can be sundried and consumed, made into liquor, or transformed into syrup, to name a few uses. Young cladodes are traditionally consumed as a vegetable, and the seeds are made into seed oil. The vast majority of the plant is edible, which only expands its beneficial horizons in regard to its environmental benefits and human health benefits.

The fruit has high levels of Ascorbic acid, Vitamin E, carotenoids, betalains, fibre, glucose, and fructose. The plant is also a natural source of antioxidants, guarding against cellular damage, and is considered a functional food by many [19].

Implementing OFI cultivars is a reality all over the world, gaining the highest importance in countries where food security is low and where fresh food is scarce during the summer months [20,21]. An unexpected result of the presence of this cactus is an increase in the soil’s health over time, allowing soils to regenerate from past malpractices, as seen in northern Morocco [22]. *Opuntia* also has a history of being an invasive species, with caution being suggested when introducing this species in an area.

This review explores the incorporation of *Opuntia* in animal feed and its benefits on soil health while the invasive dangers of the plant are also taken into account. A more chemical exploration of *Opuntia’s* ability to thrive in arid places is also made, connecting the plant’s metabolites to its abilities.

## 2. Combating Desertification and Improving Soil Health with *Opuntia*

Drylands make up 41% of the global land area, being home to more than 2 billion people spread throughout all continents. Dryland agro-systems play an essential role in sustaining these populations [23]. With the amount of helpful water on earth decreasing, the area occupied by arid soils will increase, and, therefore, it is of the utmost importance to integrate and invest in these alternative ecosystems.

Cacti are identified as a good option for plantation in these arid and semi-arid areas, as it shows high productivity and is perennial and has adaptations to water scarcity and high temperatures [24]. When compared to maize, *Opuntia ficus-indica* has 80% of its energy value, but it has the potential to produce 20–30 times more forage in the same land unit [25]. *Opuntia* fruit is considered relatively low-caloric, as is common with fruit, having 27 kcal/100 g of fresh fruit [26].

The benefits of planting cacti are not limited to only increased food security. It regulates the ecosystem by lessening the impact of wind and water erosion. The dense cactus vegetation can substantially reduce soil evaporation, and when intercropping systems are used, cacti can reduce the amount of water necessary for healthy plant growth [27,28]. It is reported that in Tunisia and Algeria, Prickly-pears are planted in contour-like patterns to maximize the efficiency of water runoff harvesting and to control soil erosion on hillslopes [29]. A similar technique is used in northern and central Africa but with a check-like pattern to prevent damage from high-intensity runoff and to prevent the inclusion and expansion of gullies [30].

A well-studied successful case of using *Opuntia* to recover depleted soils happened in Morocco regarding its native argan tree population. The argan tree is a slow-growing tree which currently provides ecological and economic support to the local population. It is an income source since argan oil, extracted from the seeds, is a staple in the cosmetic industry. Argan tree species represent 29% of the Moroccan flora, making their forest one of the most important ecosystems for Moroccan biodiversity. However, in the first half of the 20th century, this territory was highly targeted for coal, grassland, and crop exploitation, and, as a result, 240,000 ha of argan forest disappeared between 1918 and 1945.

Recent approaches in the economy and environment of Morocco have increased the area with cacti, mainly *Opuntia ficus-indica*. It has been observed that there is a positive regeneration of the Argan tree (which sprouts from its old stump) in prickly-pear orchards. In fact, the number of argan trees increased with the prickly-pear orchard’s age. This phenomenon has been explained by the increase of organic matter on the soil derived from *Opuntia*. The cactus leads to the emergence of larger and more stable aggregates in soils, which reduce the volume density of the soil, hence becoming airier and facilitating soil biological activity. *Opuntia*, in this case, works as a “nurse” plant, having a positive effect on the composition and structure of degraded soils [22].

In another part of the world, India, due to its continuously growing population, a higher demand for food is observed. Part of this country’s territory is composed of arid, desert-like regions characterized by poor soil fertility and low water. Kauthale et al. [31] studied the performance of *Opuntia ficus-indica* in these regions, recording an outstanding growth performance and biomass production. Farmers are well receiving the new crop, seeing it as an opportunity for regional food and forage security [21].

Cacti are also known for having a great potential to clean contaminated lands of environmentally hazardous pollutants [17]. The *Opuntia* root system is reported to be capable of spreading through a wide area and effectively removing pollutants [15]. It is also effective in removing petroleum-originated hydrocarbons and heavy metals from contaminated soils [32]. Nharingo and Moyo [33] used cladode extract as a green soil-washing agent to extract lead from soil. Extraction efficiency ranged from 44 to 100%, showing the potential of this plant to be used as a sustainable basis for the abatement of lead-contaminated soil.

## 3. Supplementing Cattle with *Opuntia*

*Opuntia* can be a cheap source of energy, decreasing the cost of cattle diets and being available during dry seasons or during feed shortages [1]. However, planting this species as fodder should be executed carefully due to its potential as an invasive species, which can offset its value. Studies have been conducted on the nutritional value of *Opuntia* as animal fodder and its effects on the animal’s health and growth. Using this plant to produce an innovative feed would lead to a more sustainable animal production system [15].

To feed cattle with *Opuntia*, it is essential to first characterize it and ensure its safety. Even though it is edible by humans, it could potentially cause an imbalance in the complex intestinal microbiota in ruminants or be lacking in some crucial nutrients. The low dry matter and protein content in cladodes also mean that animals must consume a high amount to satisfy their nutritional requirements [17]. Various studies, therefore, have been conducted to ensure the safe incorporation of *Opuntia* in diets. Most authors seem to agree that a diet only consisting of this plant is a risk. Therefore, the cactus is used as a supplement to the regular hay and cereal diet. Using this plant during periods of drought would also help keep animals hydrated due to the high-water content in the cladodes [34]. This would be especially helpful in regions where water is scarce, especially during the dry season.

The WANA region (West Asia/North Africa) is characterized by its harsh deserts and low water availability. Since the second half of the 20th century, the region has experienced high population growth. As a consequence, 50% of vegetation from the arid rangeland was lost due to an increase in the agricultural industry. Livestock that depend heavily on grazing on arid land vegetation have experienced a remarkable reduction of 80% of the animals. The animal’s feed requirements fulfilled by grazing are currently under 25%. Thus, animals are heavily supplemented with barley grains and other concentrate feeds. Therefore, most WANA countries have implemented measures to prevent rangeland degradation and are still seeking new solutions to do so. The search for plant species that grow in arid areas is a permanent concern, although Cactus species fit most of the requirements of a drought-resistant fodder crop. This is where *Opuntia ficus-indica* can support, being used as emergency fodder, especially during feed gaps. Animal watering is a considerable concern during summer months and drought periods, but the high moisture content in the cactus cladodes helps diminish the animal’s water needs [20].

Alhanafi et al. [35] reported 16% less water consumption in lambs fed spineless cactus cladodes compared to the control group. Feeding goats with up to 42% cactus silage was found to improve body water retention, reducing water intake without compromising animal performance [36]. In another study conducted by Firew Tegegne [37], rams fed up to 80% cactus diet showed a significant decrease in animal water requirements. The same study reported that the optimal level of substitution of the diet by cactus would be 60%. Costa et al. [5] reported that voluntary intake of water decreased with increased levels of cactus pear in the diet of lambs, reducing it from 4.9 to 2.3 kg/day of water.

Feeding small ruminants with *Opuntia* alongside other protein-rich foods such as legume residues is reported to enhance health and reproductive characteristics in livestock [38]. Menezes et al. [39] supplemented Dorper sheep with sun-dried *Opuntia* cladodes, reporting a higher digestibility and no negative impact when consuming a 36% *Opuntia* diet. This diet was also reported to be more palatable and, at the same time, to supply antimicrobial, antioxidant, immunogenic, and anticoccidial compounds. In another study conducted on adult sheep, supplying the sheep with cladodes not only lowered water consumption significantly but also incorporated it as 50% of the feed promoted weight gain [40]. Beltrão et al. [41] incorporated cladodes in the feed of lambs, reporting improved digestibility and energy production while not affecting feed efficiency. This shows that incorporating cacti while animals are in the growing stage can also be beneficial. Costa et al. [42] incorporated *Opuntia* concentrate on dairy goat’s feed and registered no difference in milk production, similar to Bispo [43], who recorded no difference in milk production when incorporating cacti into the feed of dairy cows. Ortiz Rodriguez et al. [44] fed Holstein cows a diet of 14.9% fresh *Opuntia*, which resulted in an increased quality of both the milk and cheese produced. Milk yield was also reported to have increased in the supplemented group, and an improved storage life of the animal by-products was observed, as well as reduced bacterial-colony-forming units (CFU) in the final product. These effects are not exclusive to milk but are also observed in meat quality. El Otmani et al. [45] investigated incorporating 30% of cactus in goat diets. Compared to the control, the cactus-supplemented group displayed a higher protein content and lower fat and ash.

These promising results are also not exclusive to ruminants. Pascoal et al. [46] added cacti to the diets of rabbits in the growing phase. No difference was found between the control and cactus-fed groups, including no difference in average daily consumption, weight gain, and meat quality. This suggests a safe incorporation of cacti in rabbit diets, effectively making for a cheaper feed. Another study incorporated *Opuntia ficus-indica* peel up to 15% in broiler chicken feed [47]. The inclusion of the peel led to an enhanced live body weight, feed intake, and feed conversion ratio, having no adverse effects on carcass weight or dressing percentage and showing a higher protein content in the meat. Additionally, the animals fed on a 15% diet achieved higher scores in taste tests.

It is to be noted that injuries to cattle are common when consuming *Opuntia* varieties that have spines [48]. The spines are reported to cause discomfort to the animal’s eyes, skin, and mouth, even sometimes causing gastrointestinal infections [49]. To overcome this problem, farmers burn off the spines previously or opt for spineless varieties, but these are not always available. Spiny cactus is usually cut and shredded before being given to the animals, which can be a high-effort task for farmers [50]. Another disadvantage is the short shelf life that fresh *Opuntia* has due to its high moisture content and fermentable carbohydrates. As a result, outdoor storage is only possible for a few days [51]. A way to overcome this problem is to mix the prickly pear with another dry fodder such as wheat straw. Vastolo et al. [52] concluded that the silage obtained with 5% straw is the best preserved, helping achieve a lower pH and ammonia nitrogen concentration. Another option is to dry the cactus. This drying process can take up to 14 days, depending on the thickness and size of the cladode and weather conditions [53]. Sun drying is the primarily used method as it is practical and cheap. However, removing the moisture from the plant will not result in the same desirable decreased water consumption when fed to animals. It has also been suggested that selective breeding of *Opuntia* may increase nutritional potential of its use as a fodder [20].

## 4. Invasive Potential of *Opuntia*—Strategies for Control

*Opuntia* is responsible for one of the most significant biological invasions in modern times [54]. The introduction and subsequent spread of prickly pear in Australia in 1788 caused it to infest 24 million hectares of rural land by the 1920s. *Opuntia* is so complicated and costly to control by chemical and mechanical means that enormous areas were simply abandoned by their owners. Mechanized methods such as ploughing or chopping the plan are commonly used, but at the same time, these result in accelerating the infestation of new plants that grow from broken roots and cladodes [17]. Monosodium methane arsenate and glyphosate are common herbicides used in *Opuntia* and are directly injected into the plant [55]. However, these also pose a hazard for workers and for the environment. The cactus stops endemic flora from growing by occupying land and competing with it, and it constitutes a physical barrier to animals. Protective clothing must also be worn by workers to prevent injury and irritation caused by the spines and fine glochids [17].

In 1912, natural enemies of the prickly pear were identified by a Scientific Commission created for the purpose of controlling the pest in Australia. These natural enemies included the successfully implemented cactoblastis moth (*Cactoblastis* sp.). About 2.2 billion eggs were released between 1927 and 1931. This case is often pointed to as the most successful biological pest control. However, it is essential to point out that the same cactoblastis moth is now threatening the native prickly-pear species of North America after its deliberate release to control the prickly pear in the Caribbean [54].

The use of biological control in *Opuntia* has been in wide use since the 1980s. Both *Cactoblastis cactorum* and *Dactylopus opuntiae* have seen wide success in South Africa [56]. Zimmermann and Moran [56] have stated that using a biological control is the only economically viable and sustainable way to control *Opuntia* populations.

It is important to note that *D. opuntiae*, the most-used species in biological control against the prickly pear, is considered the most disastrous pest of the *Opuntia* genus across the world [57,58]. Biological control of *D. opuntiae* has also been tested in Israel. For example, two insect predators of this species (*Hyperapsis trifucata* and *Leucopina bellula*) were released in different areas. They showed a significant role in restraining *D. opuntiae* populations. Nevertheless, further tests are needed to see the long-term efficiency and safety of this solution. Others have proposed using livestock as a means of biological control. Small cattle, such as sheep and goats, consume the fruit and cladodes after the spines are burnt off [59]. In contrast, spineless *Opuntia* varieties have higher acceptability by livestock, not needing further treatment to be eaten [60].

## 5. Metabolic and Physiological Adaptations

### 5.1. The Crassulacean Acid Metabolism (CAM) and Opuntia Productivity

Like many other arid-weather-adapted plants, the prickly pear follows a CAM metabolism, opening its stomata exclusively during the night period, thus avoiding loss of water during the day when temperatures and sun exposure are harsher and evaporation rates higher. At night, CO_2_ is fixed through the oxaloacetate and malate, accumulated in the cytosol and subsequently moved and stored in vacuoles [61]. During the day, CO_2_ is released from these molecules, and photosynthesis can proceed as a C3 plant (the most common of three metabolic pathways for carbon fixation, C3 carbon fixation occurs in all plants as the first step of the Calvin–Benson cycle), with the exception that the stomata remain closed as a way to prevent water vapour and CO_2_ escape.

*Opuntia ficus-indica* is considered an extremely productive CAM plant due to its ability to keep sequestrating CO_2_ even during prolonged drought periods, fixing large quantities of carbon in its shoot and root system [20]. An increase in the organic carbon sequestration of the soil is also observed where the prickly-pear is planted [17]. Furthermore, limiting soil and water erosion decreases the on-site loss of organic carbon [30]. *Opuntia*, being able to keep transforming CO_2_ even at high temperatures and thriving in places inhospitable for most plant species, could contribute to lowering CO_2_ in the earth’s atmosphere.

A peculiarity of C3 and CAM plants is that by increasing the partial CO_2_ pressure in the atmosphere to about 70 Pa, an increase in uptake and biomass accumulation on the plant from 30% to over 60% is observed [62]. Specifically, in *Opuntia ficus-indica*, Nobel and Israel [63] described that, in the first few months after planting in a high CO_2_ chamber, the daily CO_2_ uptake was increased by 70%, accompanied by an increase in biomass production, favouring growth. Doubling CO_2_ atmospheric concentration was also shown to enhance the activity of carbohydrate-metabolism enzymes and increase source carbohydrate production, photo assimilation transport, and sink strength [64]. After three months of exposure, glucose and starch in the chlorenchyma increased by 175% and 57%, respectively, compared to the initial value. Malate nocturnal production also increased by 75%, mirroring the changes found by Nobel and Israel [63], where an increase of 70% in CO_2_ uptake was registered.

The net assimilation rate reported by Acevedo et al. [65] was upbeat throughout the year, averaging 3.4 g per square meter per day for 1.0- and 2.0-year-old plants. This growth was shown to be seasonal, with most of the expansion occurring in the late spring to early summer months (October to January). It is also important to mention that the younger the cladodes, the higher the biomass growth, as the annual increase in the area averaged 30% for all cladodes on the youngest plants (0.4 years old) but only 3% for the oldest cladodes (4.9 years old). Therefore, due to the slow growth of older cladodes, we can conclude that most of the photosynthetic area expansion occurs through the growth of new cladodes.

### 5.2. Low Irrigation Need

*Opuntia’s* resistance to drought is in part due to its high capacity to store water in its succulent stems. As such, variations in cladode thickness can be a measure of plant dehydration and stress. When in optimal irrigation conditions, *Opuntia* sequesters water into their parenchymal tissue, becoming a recognizable mucous, thick, white layer (most of the inside of the cladode). When faced with hydric stress, this white mucous layer consistently shrinks to a measurable degree [61]. This remarkable ability of the parenchyma to store water provides the plant with an efficient buffer for many physiological responses to drought. When in severe drought, the shrinking of organs due to tissue dehydration was observed.

After two and a half months without irrigation, *Opuntia*’s acid accumulation and stomatal conductance were severely reduced, as shown in Scalisi et al. [61], although the parenchyma and chlorenchyma osmotic pressure is little affected. Moreover, plant turgor presence was also shown to be reduced by 86% compared to well-watered conditions.

In another study, Aguilar Becerril and Peña Valdivia [66] determined the physiological response of *Opuntia* to extreme and intermediate drought. It was found that, under intermediate drought, the plant’s photosynthetic machinery is stable to intermediate drought (two irrigations in a 180-day period).

Acevedo et al. [65] reported that severe water stress suppressed the nocturnal stomatal openings on mature cladodes but not on young cladodes. The characteristic acidification of the plant during the nocturnal period (due to rising concentrations of malate and oxaloacetate) was considered not sensitive to water stress.

In Madagascar, livestock keepers have long incorporated *Opuntia* into their rural economy. Assisting the cattle during the dry season is the key to successful production, and rather than looking for water through the land, pastoralists have turned to the cactus as a water source [67].

In May 2021, Madagascar was facing its worst drought in the past 40 years. The Observer Research Foundation reports that the rainfall deficit has led to massive crop failures and adversely affected livestock [68]. Attempts are being made to “climate-proof” the island’s food production, the prickly pear having a central role. The population already relies heavily on prickly-pear consumption, as most households produce or forage what they consume [69].

As climate change is a global phenomenon, climate-proofing the food supply chain is highly important, assuring food security. Therefore, we urge a start in exploration and implementation worldwide by exploring plants similar to *Opuntia*, which have a lower water consumption than conventional crops.

The presence of this cactus in the European Mediterranean region is nothing new, as it was brought to the continent by the Spanish [70] between the end of the 15th century and the beginning of the 16th century. It quickly spread to Southern Italy and Greece, where the climate accommodated the plant’s growth. Its primary use, however, was the production of the Cochineal red dye and not human consumption. The observed increase in the market value of this fruit in Europe in recent years has been driven by two factors: the shortages of water observed due to climate change and the consumers’ habits. Consumers are becoming more climate- and health-conscious and are looking for local produce and a varied diet. There is also awareness of how *Opuntia ficus-indica* requires less water and maintenance to grow, having a lower environmental impact when compared to other fruit. The plant also appeals to farmers as it is considered a “hands-off” culture, decreasing the workload and investment. More recently, the Alentejo region of Portugal has been experiencing a severe yearly drought during the Summer, making it hard for farmers to maintain their conventional crops, such as olive trees, due to the increasing watering expenses. As a result, many of these farmers have turned to prickly-pear cultivars as a far more profitable solution.

Nevertheless, higher market growth for the fruit is necessary for its more widespread plantation. Consumers in Europe are not entirely familiar with the fruit. The number of seeds, presence of spikes, and unfamiliarity with how to consume it are probably its main limiting factors in the European market. We suggest that having more processed products derived from *Opuntia* would solve these barriers with particular consideration for ready-for-consumption products.

### 5.3. High Proline Content in the Fruit and Resistance to Arid Environments

Proline is the primary amino acid found in *Opuntia’s* fruit. Other reported primary amino acids include taurine and glutamine [71,72]. Differences between samples are usually observed, which may be attributed to the maturity of the fruit, its variety, and the growth environment. According to Hernández-Urbiola et al. [73], physical conditions such as water availability, temperature, and light–dark periods are primarily implicated in protein synthesis. Furthermore, several studies have demonstrated that protein synthesis increases as cellular protection when the soil is too acidic or saline [63,66,74].

Proline hyperaccumulation is reported to accompany the extremophile characteristics of some plants, and it is believed to contribute to stress tolerance capacity [75,76]. Proline content is reported to increase when the plant is under drought conditions, high salinity, high light and UV radiation, or heavy metal or oxidative stress [77]. In addition, proline accumulating mutants of some plants demonstrate that the accumulation of this amino acid has a complex effect on the plant’s response to adverse environmental conditions [78,79].

Proline is not only able to work as an osmoregulatory molecule, but it is also able to influence stress tolerance in several ways. For example, proline has been shown to work as a molecular chaperone that can protect protein integrity and enhance the activity of enzymes [77].

Moreover, some antioxidant features have been attributed to proline. Proline has been shown to protect human cells against carcinogenic oxidative stress and to alleviate mercury toxicity in rice (*Orzya sativa*), and free radical levels were shown to be reduced in transgenic algae and tobacco plants with hyper-accumulated proline. The damaging effects of singlet oxygen and hydroxyl radicals in thylakoid membranes in plants were also significantly reduced by proline [77].

The high amount of proline found in OFI fruit when compared to other common fruits (see Table 1) may have an osmoregulatory function, protecting from the environmental stresses that the plant is exposed to. Proline could be the key to this plant’s survival in arid, sunny environments where it thrives.

### 5.4. Salt Tolerance

Recent water scarcity has restricted the availability of good-quality water for irrigation. This has driven the seeking of salt-tolerant plants that can be watered with salty water without further treatment. The search for plants with increased salt tolerance has included *Opuntia*, with studies showing that this plant has a higher-than-average salt tolerance; however, the tolerance level is very variable between varieties.

The sodium (Na) accumulation in the soil or inside the plant makes the water and nutrients required unavailable. A stress response to salinity seen in *Opuntia* is the proline accumulation, used as an osmolyte for intracellular osmotic adjustment [83]. Silva Ortega et al. [83] noted that this proline accumulation was correlated with the expression of the gene Osp5cs. Radi et al. [84] recorded that after the first week of saline stress, there is a notable decline in the levels of glycine betaine and proline. However, increasing the duration of the stress promoted the accumulation of these substances. This could be due to an adaptation period to the stress. The prickly pear already shows a high amount of proline in normal conditions, believed to help the plant survive in the semi-arid areas it inhabits. Proline accumulation is, therefore, an adaptation to protect the plant from hydric stress and saline stress.

Despite these adaptations, *Opuntia* is not entirely resistant to saline stress. Salinity irrigation of 20 dS m^−1^ (desisiemens per meter) for 90 days decreased plant height by 36.5% compared to the control and reduced cladode thickness [85]. This thickness reduction is synonymous with a decrease in water storage brought upon by the high salinity. Radi et al. [84] showed that salt stress at 15.6 dS m^−1^ reduced shoot proliferation, growth, and fresh and dry weights. Additionally, after a stress duration of 3 weeks, the survival rate was significantly decreased, being the lowest at 62.25% for remote shoots. However, explants were not affected. *Opuntia* seeds have been shown to germinate up to 45.65 dS m^−1^ but showed a low ability to recover after salt exposure, some even entering a secondary dormancy state [86]. However, a saline concentration of 3.6 dS m^−1^ was reported by Fonseca et al. [87] to not have stressed the plant. On the contrary, a 3-day interval treatment with saline water was reported to have increased plant height, the number of cladodes, cladode area index, green mass, and dry matter yield. These results directly contradict those acquired by de Lira Freire et al. [88], who applied the same 3.6 dS m^−1^ saline water treatment at a 14-day interval until the plants showed extreme chlorosis and dehydration (considered 100% damage). From the 24 genotypes studied, the least resistant was the *F-8* variety, showing 100% damage at 130 days, and the most resistant variety was *Liso forageiro*, surviving 419 days.

Salinity already present in the soil may influence the outcome of salinity tests. We must point out that, from the studies made on soil, only de Lira Freire et al. [85] measured the actual soil salinity. The other studies simply irrigated the plant with a known salinity solution.

### 5.5. Proline, Indicaxanthin, and Proline Betaine

**Figure 1:**
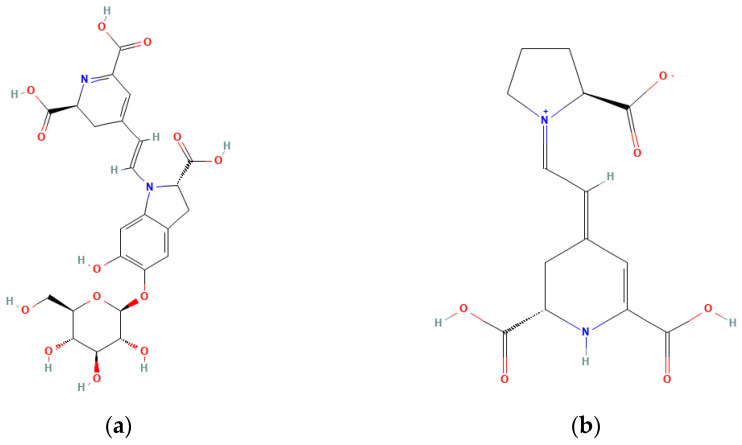
Structure of (**a**) betanin and (**b**) indicaxanthin [93,94].

Betalains such as betacyanins and betaxanthins are rare in the human diet, being only significantly present in beets and prickly pear [89]. Different betalains (Figure 1) are detected in *Opuntia,* with indicaxanthin being usually the dominant one [89,90,91,92], although it mostly depends on fruit variety.

Indicaxanthin has a yellow colouration, while betanin has a red colouration. As such, depending on the fruit variety (which have different colourations), the relative amounts of these two pigments differ. In red varieties, betanin is prevalent, while in yellow varieties, indicaxanthin predominates (see Table 2).

Indicaxanthin is derived from L-proline instead of tyrosine. Unlike anthocyanin, commonly found in plants, betalains contain nitrogen, as they are aromatic indole derivatives synthesized from tyrosine. *Opuntia* shows much higher levels of proline compared to tyrosine. We can hypothesize that the high amount of proline resulted from the high indicaxanthin content, or the plant accumulates proline for indicaxanthin synthesis.

Physiologically, indicaxanthin is a very stable molecule that is considered a significant antioxidant. In vitro, tests point to its ability as a radical scavenger and protector against cytotoxicity by various agents [96].

**Figure 2:**
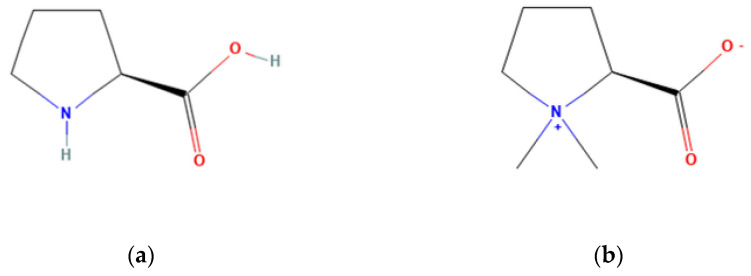
Chemical structure of (**a**) proline and (**b**) proline betaine [100,101].

Another compound, proline betaine, is produced by the methylation of proline in plants (See Figure 2). This molecule has not been assessed in the *Opuntia* plant. In alfalfa plants (*Medicago sativa*), it was shown that high salinity originated a 10-fold increase of proline betaine in the shoots [97], suggesting an osmotic adjustment role. This protective role of proline betaine, however, remains controversial, as not all plant species produce this compound and introducing it to a plant does not always lead to an increased environmental stress tolerance [98] Genetic engineering has been used to produce a higher amount of Proline Betaine in *Citrus*, resulting in an increased stress resistance as described in Nolte et al. [99]. Since a high amount of proline is naturally found in *Opuntia* and betaine, it can be hypothesized that proline betaine could also be present and have a role in the adaptations to unfavourable conditions of this plant.

## 6. Conclusions

*Opuntia* displays a high potential for future usage as water availability lowers and average temperatures rise. Its adaptation to hot, dry weather assures its growth in harsh conditions. Furthermore, its usage includes food and fodder for cattle, a water reserve, and a safe decision for soil health.

Considering the expected climate changes, with increasing temperatures and water scarcity in some regions such as the Mediterranean basin, North Africa, and South Asia, *Opuntia* can contribute partially to the solution, whether in assuring food security or lessening water consumption or decreasing the desertification of soils.

## Figures and Tables

**Table 1:** Comparison between the proline found in common fruit juices and *Opuntia’s* fruit (Prickly-pear). Data for different *Opuntia* varieties are also included [71,80,81,82,83].

Fruit	Proline (mg·L^−1^)	
Prickly pear “*Morado*”	883.4	[71]
Prickly pear “*Gymno Carpo*”	1143.5	[71]
Prickly pear “*Apastillada*”	1768.7	[71]
Orange	436	[70]
Grape “*Airen*”	122.03	[80]
Apple “*Golden*”	45.5	[81]
Pomegranate	231	[82]

**Table 2:** Amount of betanin and indicaxanthin in the prickly pear pulp (mg/100 g of edible pulp) [95].

Fruit Variety	Indicaxanthin	Betanin
Yellow	8.42 ± 0.51	1.04 ± 0.12
Red	2.61 ± 0.30	5.12 ± 0.51
White	5.86 ± 0.49	0.10 ± 0.02
